# GPCRs as Partners of LGR5 Cancer Stem Cells: Opportunities and Challenges

**DOI:** 10.3390/biom16101522

**Published:** 2026-10-19

**Authors:** Jeetendra K. Nag, Ganesh Subedi, Rachel Bar-Shavit

**Affiliations:** 1 Department of Heart, Blood and Kidney Research, Cleveland Clinic Research, Cleveland, OH 44195, USA; nagj3@ccf.org (J.K.N.); 2 Department of Chemistry, Cleveland State University, Cleveland, OH 44115, USA; subedig@ccf.org; 3 Sharett Institute of Oncology, Hadassah-Hebrew University Medical Center, Jerusalem 91120, Israel

**Keywords:** cancer stem cells (CSCs), G protein-coupled receptors (GPCRs), leucine-rich repeat-containing GPCR 5 (LGR5), β-catenin stabilization

## Abstract

Hierarchical organization transpires within a tumor. It is appreciated that cancer stem cells (CSCs) are the cornerstone primary cells of such assemblies, fueled by molecular mechanisms that allow resistance to chemotherapy, vigorous self-renewal capacity, and resistance to immune therapy. Due to their powerful self-renewal capability, CSCs constantly initiate tumor cell proliferation, maintaining the growth state of the tumor. Leucine-rich repeat-containing G protein-coupled receptor 5 (LGR5), a known stem cell niche protein, is a G protein-coupled receptor (GPCR) that is involved in Wnt signaling. It was previously shown that members of protease-activated receptors (PARs) and another affiliate of GPCRs, the G protein-coupled receptor 25 (GPR25), elicit β-catenin stabilization and transcriptional activity. PARs and GPR25 can associate with LGR5 and markedly enhance β-catenin stabilization. The prospect of identifying additional cancer driver GPCRs partners of LGR5, such as the prostaglandin receptors, EP2 and EP4, and the lysophosphatidic acid receptors (LPARs), which have been demonstrated as capable of inducing β-catenin stabilization yet pending experimental association with LGR5, is discussed. The existence of an LGR5-GPCRs-β-catenin hub may potentially overcome tumor plasticity and alleviate drug resistance. It certainly elucidates new opportunities in the fight to eradicate cancer.

## 1. Introduction

The study of cancer stem cells (CSCs) has become a cornerstone for understanding cancer biology. The basis of the CSC-based concept is that hierarchical organization takes place within a tumor, implying that cells in a tumor are not equal but rather display considerable heterogeneity. It is well appreciated that CSCs may evade anti-tumour immunity and exhibit resistance to immunotherapy, a capacity for vigorous self-renewal and resistance to immunotherapy [1,2]. Due to their powerful self-renewal capability, CSCs constantly initiate tumor cell proliferation, thereby maintaining the tumor growth state. Accordingly, the CSC model has stimulated innovative treatment strategies targeted at specifically terminating CSCs, rather than general shrinkage of the tumor mass.

Another important aspect of CSCs is their plasticity, appointing dynamic capability to transfer back and forth between stem-like statuses and differentiated non-stem positions, directing therapy modalities of metastasis, resistance, and tumor relapse. In addition, CSCs resist standard treatments through basic cellular mechanisms and signals from the surrounding microenvironment, making them a primary driver of cancer relapse [3,4]. Importantly, CSCs direct therapy resistance, tumor growth, and metastasis, while depending greatly on bidirectional cross talk with the surrounding tumor microenvironment (TME) [5].

It should be noted that stem cells do not exist only in cancer. Normal physiological adult stem cells, also called somatic stem cells, are found in various tissues. These stem cells are involved in tissue regeneration and repair [1,6,7].

Lineage tracing analyses have enabled the detection of CSCs and recreation of their history, starting from their tumor origins. Due to the high degree of plasticity exhibited by cancer cells, studies in both mice and humans have shown that the bulk of cancer cells that disseminate and metastasize do not express leucine-rich repeat-containing G protein-coupled receptor 5 (LGR5). Nevertheless, conversion of such cells into LGR5-expressing cells is essential for the colonization status of metastatic foci [8,9]. In colorectal cancer, for example, lineage tracing has assigned LGR5 as being highly expressed at the origin of the tumor. LGR5 is a seven-transmembrane domain cell surface receptor and a member of the G protein-coupled receptor (GPCR) family (although it does not act via heterotrimeric G proteins). LGR5 is a Wnt target gene expressed at the crypt base of the small intestine [9,10,11,12]. While previously considered an orphan receptor, LGR5, along with its homologs LGR4 and LGR6, form a family of receptors that are activated by R-spondins [13,14]. LGR5-expressing adult stem cells are abundantly present in the intestinal epithelium, residing in the intestinal crypt and comprising up to 10% of colorectal cell population [15]. More precisely, these LGR5-negative crypt base columnar (CBC) stem cells are scattered among Paneth cells at the base of the crypts. It is also known that LGR5 plays a role in drug resistance development, based on studies demonstrating that LGR5-expressing cells become non-LGR5-expressing cells that show drug resistance and a non-proliferative status upon chemotherapy [16,17]. The high degree of plasticity that exists in advanced tumors is reflected by the fact that when LGR5 cells are depleted, LGR5-negative cells can become LGR5-expressing cells that replenish adult intestinal crypts, thus restoring and maintaining tumor hierarchy [8]. Significantly, although LGR5 is a primary CSC indicator, therapies targeting LGR5 alone with the aim of eradicating tumors are not sufficient to eliminate a cancer.

Clinical and preclinical indications show that, in fact, targeting LGR5 alone is unsatisfactory to eradicate cancer. Lineage-tracing models indicate that while tumor spread is driven by LGR5-negative cells to successfully colonize metastatic lesions at distant organs, these cells must express LGR5. Hence, targeting LGR5 alone fails to address driver genes involved in the spread and migration of LGR5-negative cells [18,19].

Another aspect is the observation that loss of LGR5 increases therapy resistance. Studies have demonstrated that upon neutralization of LGR5, either via gene ablation or via LGR5-targeted antibody drug conjugates, in response to chemotherapy, led to increased levels of STAT3. Activation of STAT3 caused increased MET levels, which were reversed in the presence of LGR5. This also takes place vice versa: a decreased STAT3–MET axis improved sensitivity to chemotherapy. Based on these xenograft observations, the application of STAT3 inhibitor in the presence of irinotecan improved suppression of tumor growth and prolonged survival [18]. One must keep in mind that LGR5 is not only a marker of cancer stem cells, but also adult epithelial stem cells, such as in the human stomach, gut, and hair follicles. Depletion of LGR5 may have the risk of destroying the niche of healthy epithelial stem cells, leading to gastrointestinal toxicity and affecting tissue regeneration [8,15,20].

Being replaced every several days, the intestinal epithelium is the most dynamic tissue among mature mammals that is repeatedly self-renewed. The constant proliferation activity within the crypt of the villi is driven by LGR5-expressing cells, producing progenitor stem cells. Among the driving forces implicated in intestinal tract cancer is the Wnt/β-catenin pathway, activated upon ligation of Wnts and the subsequent activation of Frizzled (FZD) receptors. Certainly, FZD receptors play a key role in cancer growth and progression, being negatively regulated by homologs E3 ubiquitin ligases, namely, ring finger 43 (RNF43), and its homolog, zinc and ring finger 3 (ZNRF3). These ubiquitin ligases function as negative controls of the Wnt signaling pathway, acting by tagging FZD receptors with ubiquitin, thereby assigning them for lysosomal degradation [21]. Counteraction of this negative regulation relies on the binding of R-spondin to LGR5, which enables stem cell release and rescues FZD receptors from degradation. LGR5-R-spondin promotes internalization of the negative regulators ZNRF3/RNF43, thus leaving cell surface FZD receptors functionally active [21].

GPCRs are the largest family of cell-surface molecules that transmit a signaling web across the cell membrane. Analysis of the Cancer Genome Atlas (TCGA) has primarily redesigned our comprehension of GPCRs in cancer. While they have been overlooked as oncogenes, TCGA multi-omics analyses show that GPCRs are extensively mutated, dysregulated, and differentially expressed across a wide range of human tumors. The aberrant overexpression of membrane GPCRs designates, by far, the most frequent mean used by tumor cells, followed by ligands secreted by tumor cells or the microenvironment to activate the abundant GPCRs and initiate signaling networks. Less frequent are occasions of mutations in GPCRs and G proteins, as well as copy number variations. This was shown by using integrated analysis of mutations, copy number variations, and differential expression of GPCRs in cancer [22,23].

Although GPCRs in cancer are somewhat neglected, as reflected by the few GPCR-based cancer therapies, their importance in cancer biology is now emerging. Traditional GPCR signaling depends on specific activation of G-proteins, yielding a range of signaling circuit repertoire termed “biased agonism.” GPCRs can also act independently of heterotrimeric G-proteins, as best demonstrated by FZDs, corresponding to Wnt receptors, which can act independently of G-proteins. LGR4-6 GPCR family members are also considered to act independently of G-proteins upon ligation by R-spondins, acting as part of the Wnt/β-catenin pathway. Analysis of TCGA for GPCR-linked criteria, such as mRNA level of expression, copy number variations, and mutations, indicated that all these traits are found in cancer-involved GPCRs [20,21]. Indeed, many GPCRs are overexpressed in solid epithelial tumors, contributing to tumor growth and development. Among the GPCRs that are overexpressed in cancer are the protease-activated receptors (PARs) (Table 1 [24,25,26,27,28,29]), EP2, EP4 prostaglandin receptors [Table 1; 32–40], and receptors for lipid activators, such as lysophosphatidic acid receptors LPARs [Table 1; 39–46] and sphingosine phosphate (SIP) (Table 1 [30,31]). These GPCRs can induce nuclear β-catenin localization and transcriptional activity. Therefore, they serve as good candidates for cancer driver genes involved in cancer stemness (Table 1).

The tumor microenvironment (TME) is a flexible dynamic environment in which both soluble and matrix-immobilized proteases are engaged to maintain tumor progression [49]. Indeed, one of the goals for maintaining intestinal health is to better understand proteolytic homeostasis that occurs at the mucosal surface. Proteases in the gut, either derived from the host or microbiota, are actively engaged in a wide range of physiological and pathophysiological processes. Dysregulated proteolysis (dysbiosis) leads to gut disorder-related events, such as barrier dysfunction, remodeling of the matrix microenvironment, inflammation, and cancer. While extracellular proteases convert inactive pro-forms into active forms via appropriate cleavage or act to degrade proteins, they also function as signaling proteases. Here, we focus on signaling proteases stored and anchored at the TME and extracellular matrix (ECM) in promoting powerful cross talk via the activation of PARs, which are abundantly expressed on nearby tumor cells.

Considering GPCRs as partners in stem cell functions is appealing. Still, the molecular mechanism of GPCR involvement remains understudied.

LGR5 is acknowledged as a cancer stem cell protein and, as such, a significant cancer drug target. Nevertheless, addressing LGR5 alone is not enough to eradicate cancer due to the high degree of cell plasticity, whereby cells that do not express LGR5 often become LGR5-positive cells. It is therefore essential to detect and allocate collaborators that co-associate and act with LGR5 to better eradicate metastatic colonies.

LGR5 and GPCR partners that can induce β-catenin stabilization might form a suitable platform for drug design that opens new possibilities for cancer therapeutic regimens.

The aim of the present review is to put forward GPCRs as potential regulators involved in the CSCs niche. Here, we illustrate via a roadmap introducing CSCs hierarchy in cancer, followed by the Wnt/β-catenin pathway, including LGR5 function as a marker of stem cells and a target of the β-catenin stabilization path. Next, we discuss the part taken by GPCRs in cancer. Subfamilies of GPCRs and members of PARs, PAR_2_ and PAR_4_, as well as GPR25, can induce β-catenin stabilization and co-associate with LGR5. Based on this, we propose that these GPCRs may function as partners in stem cell niche events. It is further suggested that additional cancer GPCRs, such as EP2, EP4, and LPARs that induce β-catenin stabilization, are potential candidates, pending experimental validation that they are involved in the CSC niche.

## 2. LGR5 Potentiates Wnt/β-Catenin Signaling

Once LGR5 is activated by R-spondins, it potentiates Wnt/β-catenin signaling, contributing to CSC self-renewal and tumor growth [50,51]. Stabilization of β-catenin and its nuclear transcription activity are initiated by the primary formation of a Wnt-FZD-LRP (low-density lipoprotein receptor-related protein) complex following Wnt binding to FZD receptors at the cell surface and LRP5/6 co-receptors. This leads to induced expression of a gene signature that controls stem cell traits. To date, 10 mammalian FZD receptors, 19 Wnt lipoproteins, and two LRPs (LRP5 and LRP6) have been described. Since Wnts bind to a conserved cysteine-rich extracellular domain of FZD receptors, there is seemingly no specification for the association of a certain Wnt with a FZD receptor. Rather, Wnts can bind to several FZD receptors and LRPs. The tumor microenvironment is a rich source of Wnts secreted from cancer-associated fibroblasts (CAFs) [51,52] and immune cells [52] capable of activating tumor cells [53]. Formation of a Wnt-FZD receptor-LRP complex initiates a cascade of events at the inner leaflet of the cell membrane, recruiting the scaffolding signal protein Dishevelled (DVL), which then dissociates from the “destruction complex.” In fact, the destruction complex comprises of casein kinase 1α (CK1α), adenomatous polyposis coli (APC), axin, and glycogen synthetase kinase-3 (GSK-3), which once dismantled, allows the stabilization of β-catenin and its nuclear transcription activity [54,55].

Ring finger protein 43(RNF43) and zinc and ring finger 3 (ZNRF3) are transmembrane RING-type E3 ubiquitin ligases that function mainly as tumor suppressors by negatively controlling Wnt/β-catenin signaling. These E3-ligases ubiquitinate Wnt receptors like Frizzled (FZD) and LRP5/6 co-receptors on the cell surface, leading to endo-lysosomal degradation and turning off the Wnt/β-catenin signaling. Rescue from the negative RNF43/ZNRF3 regulation of FZD receptors unravels the association of a LGR5-RSPO complex with E3-RNF43, leading to the formation of a LGR5-RSPO-RNF43 trimer at the cell membrane, which leads to inactivation and internalization of RNF43, thus no longer capable of negatively regulating FZD receptors [56,57,58]. As a result, the degradation of FZD receptors is halted, leading to elevated Wnt receptor levels and, consequently, enhanced β-catenin signaling [6,59]. However, how LGR5 potentiates Wnt/β-catenin signaling is under dispute. For example, studies by the Liu group showed that LGR5 interacts with both FZD receptors and LRP6 and induces LRP6 phosphorylation, thereby boosting Wnt-β-catenin signaling [60].

Molecular patterning of LGR5 in the mouse intestinal tissue indicates co-expression with tissue sections undergoing Wnt-induced proliferation, indicating its essential role in preserving crypt architectures [13]. In parallel, conditional deletion of genes encoding both LGR5 and Wnt causes instant damage to the intestine crypts, much as obtained when the Wnt pathway is inhibited. Taken together, these observations point to an intimate relation between LGR5, the adult stem cell protein, and FZD receptors. In fact, all members of the R-spondin family that activate the Wnt signaling pathway act in combination with Wnt to induce β-catenin stabilization [61,62,63]. While deletion of the R-spondin 1 (RSPO1) gene led to the destruction of mouse intestinal crypts, these structures can be repaired in the presence of Wnts. Studies from the Clevers group using mass spectrometry and bait capture analyses demonstrated a direct association between LGR5 and FZD receptors. Affinity purification analyses using LRG5 as bait captured FZD5, as well as FZD6, LRP5, and LRP6. Co-association of FZD receptors and LGR5 was also demonstrated by co-immunoprecipitation (co-IP), fluorescence resonance energy transfer (FRET), and immunofluorescence approaches [13]. The FZD receptor–LGR5 association provides a critical guiding regimen for therapy design aimed at inhibition of LGR5 and R-spondin in hyperactive Wnt-driven cancer.

## 3. Telocytes in the Intestinal Tract Express LGR5

The structured organization of intestinal epithelial cells includes crypt-villi components organized in a repetitive manner. While considering CSCs and LGR5 association with cancer driving partners, the localization of LGR5 stem cells throughout the intestine is of interest. LGR5-expressing stem cells and Panet cells constantly divide to generate proliferative progenitors at the base of the crypts that drift upstream toward the villi. At the miRd-villus, one finds transit-amplifying cells, and the tip of the villus is occupied by terminally differentiated cells. While cells at the tip of the villus continually shed into the lumen area, they are rapidly replaced by cells from the crypt base [11,15]. Another type of cells that accompanies and impacts intestinal epithelial cells are telocytes. Telocytes, distinctive cells expressed in many tissues, including the intestine, are mesenchymal cells characterized by long and extensive cytoplasmic extensions named telopodes. These extensions form a set of connections with other cell types and are recognized as playing important roles in the intestine stem cell niche [64,65]. Recently, a new location for LGR5 was demonstrated. Using laser capture micro-dissection and single-cell transcriptome analysis, Bahar Halpern and colleagues demonstrated that villous tip telocytes express LGR5, previously referred to as a crypt base stem cell indicator [65,66]. Ablation of these villi tip LGR5-expressing cells perturbs and inhibits genes that are central to the intestinal structure program [65,66]. The full implication of villi telocyte LGR5-expressing cells remains to be defined. While crypt-villi epithelia are constantly dividing and persistently being turned over, telocytes rarely divide and instead are quiescent cells. These telocytes may thus serve as memory entities that can direct and contribute to the activation of the LGR5 crypt-villi program.

## 4. LGR5-IQGAP1 Interactions

Traditional GPCR signaling is mediated by binding to G-proteins, which facilitate the switch between the GDP- and GTP-bound states. One G-protein family includes small guanine nucleotide-binding and hydrolyzing proteins (21–25 kDa) that are activated only when bound to GTP, such as Rho, Rac, and Cdc42, that control focal adhesion complex formation, cytoskeletal actin assembly, filopodia construction, and membrane ruffling, respectively [67,68]. IQGAP1 (IQ motif-containing GTPase-activating protein 1) is a 190 kDa protein that contains several protein-interacting domains and binds the GTP-bound active forms of Rac and Cdc42 in vivo, rather than GDP-bound forms, stabilizing their active state [69]. IQGAP1 is a scaffold protein that combines cellular signals regulating the actin cytoskeleton, cell adhesion, and events of the cell cycle. Discussing GPCRs potential interactions with LGR5, this mode of GTPase switches binding is part of GPCR signaling. Interestingly, LGR5 can associate with IQGAP1 to promote Rac/Cdc42 binding to enhance cell–cell adhesion and actin reorganization [69,70,71]. This demonstrates how LGR5 not only associates with GPCRs but can also connect with G-protein switches for signaling.

## 5. PARs Co-Associate with LGR5

The TME and ECM act as bioactive depot sites as dynamic storage reservoirs that actively sequester soluble, cell-secreted, and ECM-immobilized proteases [72,73]. Mammalian PAR family members provide a good paradigm that exemplifies the robust cross talk between proteases residing in the TME and their cancer cell surface receptors. PARs form a subfamily of GPCRs comprising four members, PAR1-4, encoded by *f2r*, *f2rl1-3*, respectively [74]. The extracellular portion of PARs contains their ligands (specific for each PAR member), capable of activating PARs following appropriate cleavage to unmask their hidden ligands. We previously demonstrated PAR_1_-induced stabilization of β-catenin in a transgenic mouse line overexpressing PAR_1_. In these mice, PAR_1_ is overexpressed in the mouse mammary fat pads. High levels of β-catenin are obtained in transgenic mouse mammary glands overexpressing PAR_1_, as compared to their wild-type counterparts [75]. In addition, we identified pleckstrin-homology (PH)-binding domains critical for cancer growth and development within the cytoplasmic tails of PAR_1_/*f2r*, PAR_2_/*f2rl1* [76,77,78], and PAR_4_/*f2rl3*. These sites can connect with cancer signal proteins via their PH domain, among which are Akt, Etk/Bmx, Vav3, Gab1, and Sos1, all of which have been demonstrated to directly bind and associate with PARs. Since the PH-binding domains within the C-tails of PARs are essential for cancer growth and development, we generated, toward PAR_2_ and PAR_4_ PH-binding motifs, a library of peptides. With the intention of developing these findings toward clinical application, we selected a lead cyclic peptide named P*c* (4-4) with the aim of attenuating PAR-induced signaling as a future cancer drug therapy. P*c* (4-4) effectively inhibits epithelial tumor growth in vivo, as demonstrated with colon, breast, and ovarian cancer xenograft mouse models [75,76]. Significantly, P*c* (4-4) also inhibits PAR_4_-induced activation of EGFR/erbB2, as recapitulated by the inhibition of phospho-tyrosine (pTyr) EGFR1 [78]. Therefore, PAR inhibitors may also become beneficial drugs for Her2/*Nue* breast cancer patients who may develop resistance, for example, to treatment of Herceptin.

It is well acknowledged that PAR_1,2&4_ GPCRs play driving roles in tumorigenesis [75,76,77,78,79,80,81,82], mediated in part by PAR_1,2&4_-induced β-catenin stabilization and its nuclear transcription activity. PAR-induced β-catenin stabilization takes place effectively in the presence of Wnt/FZD receptor pharmacological inhibitors [79]. PAR_2_ induces potent β-catenin stabilization under conditions where Wnts are inhibited, i.e., in the presence of Porcupine (e.g., LGK974) or the presence of FZD receptor inhibitors; sFRP (secreted Frizzled-related protein) binds the extracellular portion of FZD receptors at the Wnt-binding site, thus neutralizing FZD receptors. To date, primary regulators that control the PAR-induced β-catenin path have been understudied. Gaining insight into the posttranslational regulation of PAR_2_ involvement in colon cancer progression was obtained by demonstrating the negative role of RNF43 in a PAR_2_-induced colon cancer setting. RNF43 ubiquitinates PAR_2_, leading to PAR_2_ internalization and degradation. PAR_2_ degradation is reversed in the presence of LGR5 and R-spondin. This mode of PAR_2_ regulation is found to be comparable with FZD receptor RNF43-mediated mode of degradation and rescue [79].

**Figure 1:**
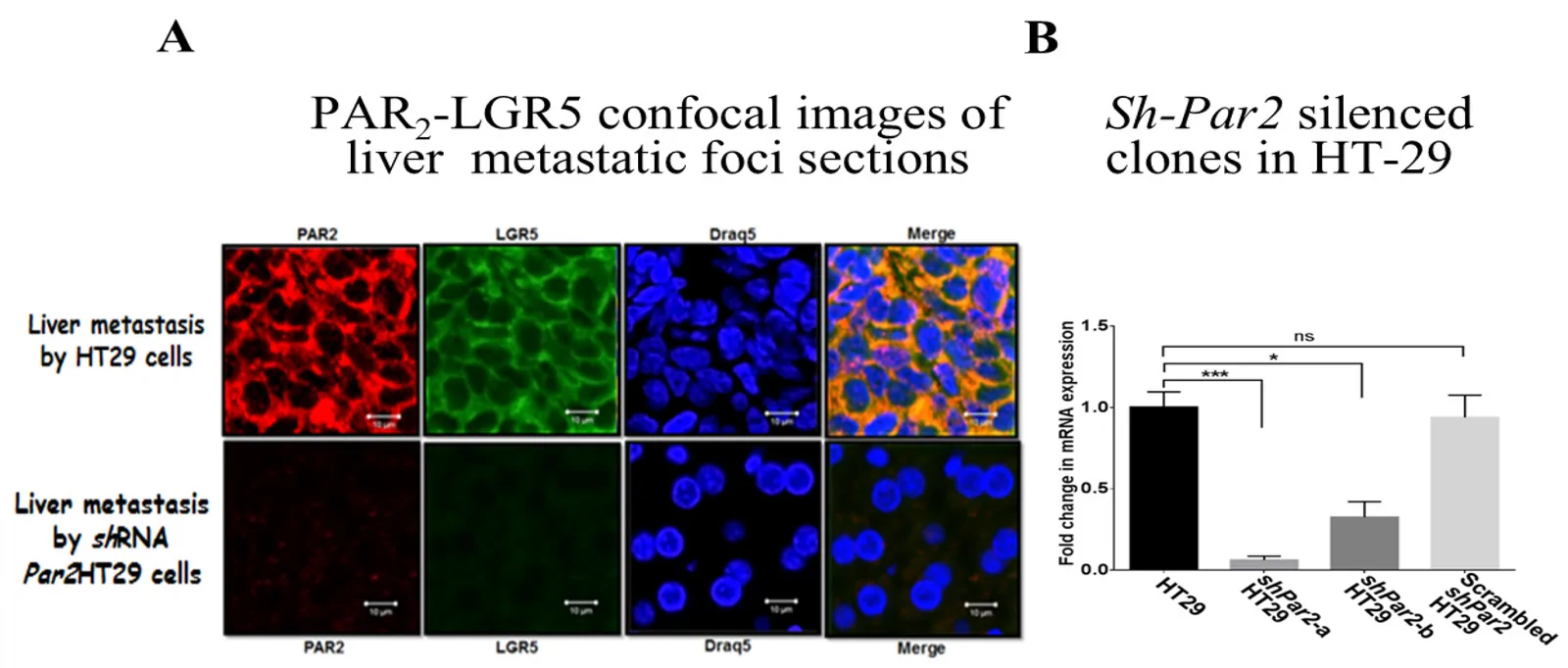
(**A**). PAR_2_-LGR5 confocal image of liver metastatic foci sections. Co-localization of PAR_2_ and LGR5. Fluorescent images of liver sections taken following intrasplenic orthotopic inoculation of HCT116 cells-derived metastases. Scale bar: 10 μM. Abundant levels of PAR_2_ (red) and LGR5 (green) are seen in the livers of mice undergoing PAR_2_-driven metastasis. Strong co-localization (orange) of PAR_2_ and LGR5 is seen. No to very little expression of either PAR_2_ or LGR5 is seen when *sh*RNA-*hPar2*-silenced HCT116 cells were similarly injected into the spleen. Nuclei were stained blue following detection by Draq5 [79]. (**B**). *shPar2* silenced clones of HT-29. qRT-PCR evaluation of *Par2* expression was carried out in clones of *shPar2* silenced HT-29 cells. * Statistical significance *p* < 0.05; *** statistically significant *p* < 0.001, ns—non significant.

Interestingly, as found for FZDs association with LGR5, it also co-associates with PAR_2_ (Figure 1). LGR5-PAR_2_ potently enhances PAR_2_-induced β-catenin stabilization and TOP*flash* transcriptional activity [81]. The association between PAR_2_ and LGR5 was revealed by co-IP and immunofluorescence (Figure 1 and [79]) and co-IP. These properties assign PAR_2_ as possibly involved in regulating the CSC niche.

The PAR_2_-LGR5 platform provides a useful hint as to the potential benefit of PAR-based therapy for cancer. Self-renewal capacity of CSCs was originally established by O’Brein CA et al. [80], demonstrated by the limited dilution assay (LDA) in a xenograft mouse model. The assessment for self-renewal by *Par2*, in vivo, was evaluated, granting *Par2* with CSC properties. For this purpose, ectopic inoculation of HCT116 cells, expressing high levels of *Par2* versus silenced *Par2*, shRNA-*Par2*-HCT116 cells, was employed. Inoculation of 1000 HCT116 cells gave large tumors. Consistently smaller tumors were seen in the *sh*RNA-silenced *Par2*-HCT116-injected cells, as minimally as 10 HCT116 cell numbers can initiate tumors, which was completely abolished by *sh*RNA*-Par2*-silenced HCT116. Histograms of mouse tumor volume. ** *p* < 0.001, * *p* < 0.05 [79].

**Figure 2:**
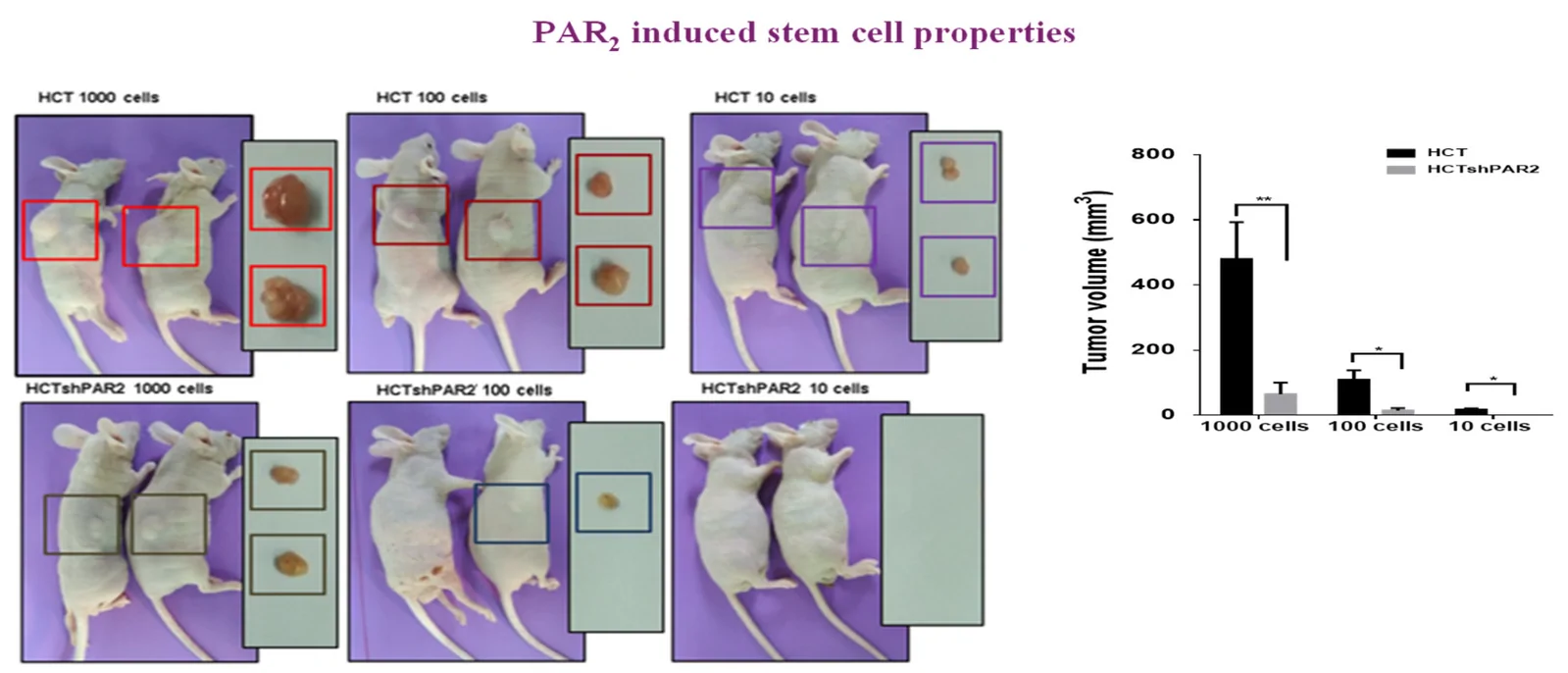
(**Left**). LDA of HCT116 cell versus *sh*RNA*Par2*-HCT116 cells. Serial dilutions of cells in mouse xenograft model, in vivo. Serial dilutions of *wt* HCT116 and *sh*RNA-*Par2*-HCT116. Initiated with 1000 cells and down to 10 cells, inoculated subcutaneously. A minimal number of 10 cells of *wt* HCT116 generated a tumor yet silenced *sh*RNA *hPar2* tumors. (**Right**). Histogram indicating tumor volume. Histograms of mouse tumor volume. ** *p* < 0.01, * *p* < 0.05 [79].

There was also a lower number (decreased by ~6-fold) of tumor initiations in the *sh*RNA-*Par2*-silenced HCT116, proposing that *Par2* is a member of colon cancer stem cell niche (Figure 2).

**Figure 3:**
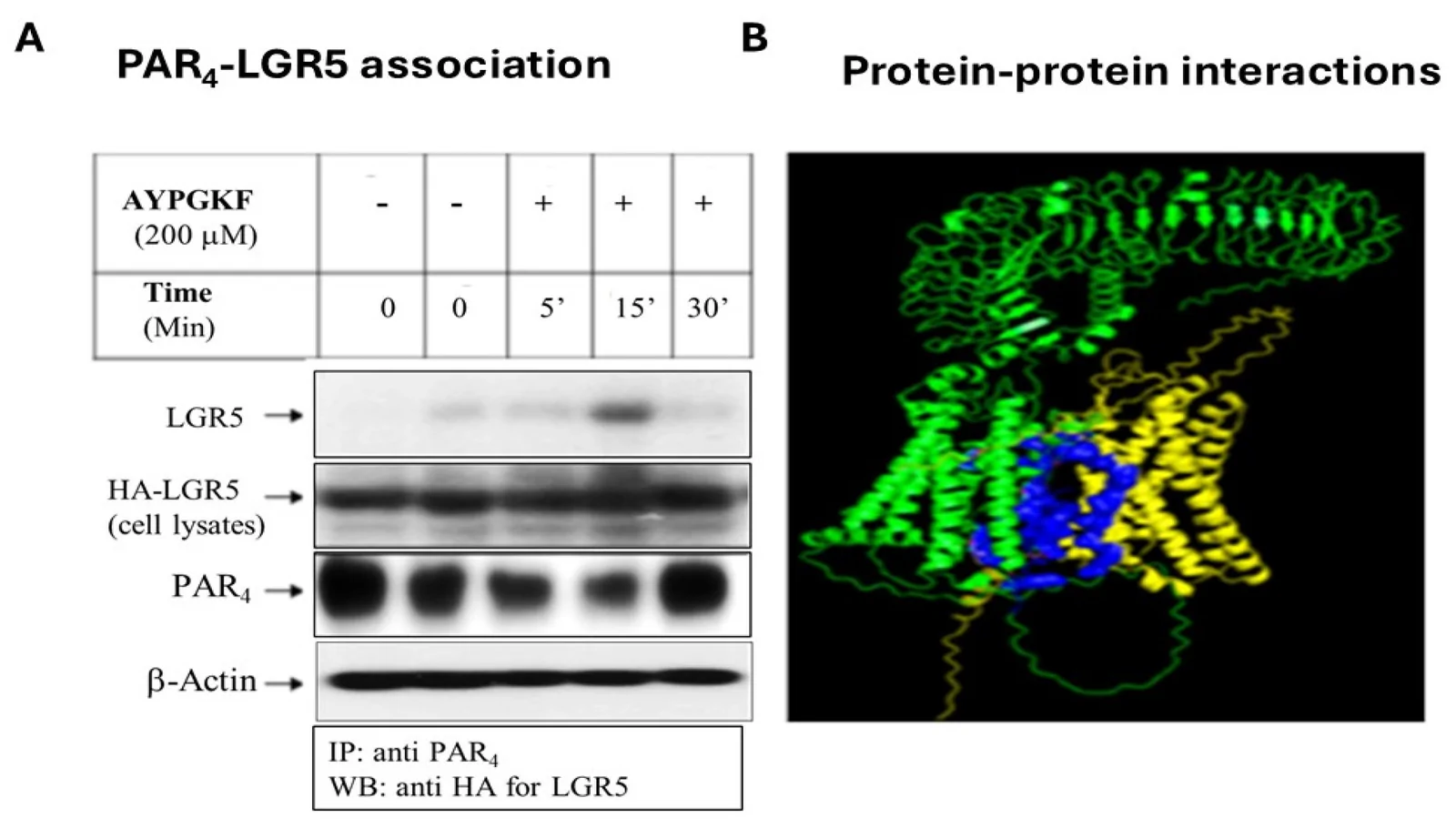
PAR_4_-associated LGR5. (**A**) PAR4-LGR5 complex formation. PAR_4_ forms a complex with LGR5 following AYPGKF PAR_4_ activation. (**B**) Protein–protein docking. PAR_4_ (yellow) and LGR5 (green) interaction was predicted using AlphaFold 3 and HADDOCKv2.4. The blue region represents the predicted interface between the two proteins [82].

The role of PAR_2_ as a stem cell marker in colon cancer was also assigned by Ma Y et al. [81], who demonstrated the upregulation of the stem cell transcription factors SOX2, Nanog, and KLF4, as well as an increased ratio of CD133/CD44 stem cell markers that together indicate resistance to therapy and poor prognosis. PAR_4_, another member of the PAR family, was shown to be capable of markedly inducing β-catenin levels and transcriptional activity. Likewise, PAR_4_ co-associates with LGR5, as evaluated by co-IP and protein–protein docking analyses (Figure 3; [82]).

With respect to protein–protein docking analyses, it should be noted that a growing number of studies have reported the presence of homo- and heterodimers and even higher-order oligomers following GPCR activation [83]. The role of GPCR dimers was confirmed by advanced fluorescence methods, such as FRET and bioluminescence resonance energy transfer (BRET), which enabled the concurrent measurement of GPCR signaling and aggregation. A docking assay for transmembrane (DAFT) components enabled massive, automated testing of different GPCR dimerization interfaces [84]. These methods indicated that for dimerization to occur, GPCR transmembrane domains come together through lateral diffusion within the lipid bilayer [85,86].

**Figure 4:**
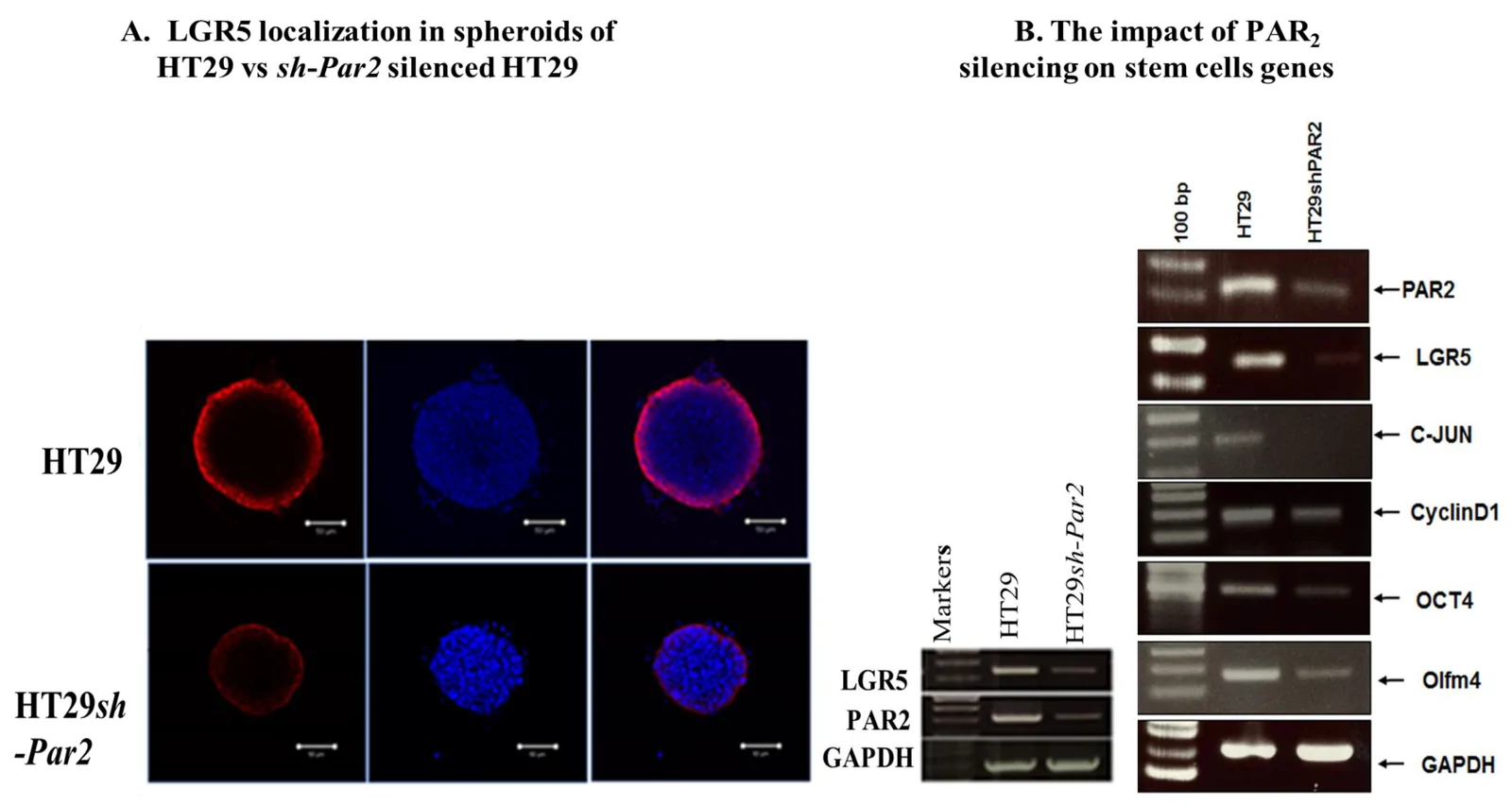
*shPar2* knockdown leads to decreased *lgr5* expression. (**A**) Immuno-fluorescence. LGR5 immuno-fluorescence in HT29 cells inhibited *Par2* expression. Scale bar: 50 μM. Nuclei are in blue and LGR5 is in red. (**B**) RT-PCR of the PAR_2_ target gene signature in wild-type and *sh-Par2*-treated HT-29 cells [79].

A further point of interest addressing *Par2* and *lgr5* is data showing that the *shPar2* knockdown of *f2rl1* in HT-29 colon cancer cells resulted in the inhibition of *lgr5* RNA levels (Figure 4A,B). This was reversed in the presence of *f2rl* [79]. One explanation for this observation is that once the silencing knockdown of *f2rl* takes place, essential transcription factors and/or other co-factors that are required for *lgr5* expression are depleted. This does not, however, exclude additional alternative reasons, which need to be fully studied.

## 6. GPR25-LGR5 Interaction

GPR25 is a poorly investigated seven-transmembrane GPCR of unidentified function. GPR25 was discovered in 1997 [87] and considered an orphan receptor of no known ligand. Recently, CXCL17 chemokine was assigned as an activator of GPR25 [88]. Yet the continuing uncertainty surrounding the endogenous ligand of GPR25 is ongoing. Data from the Human Protein Atlas revealed that GPR25 is predominantly expressed in the stomach and lung at the tissue level and in some immune cells [88,89]. GPR25 presents disulfide bonds linking residues C112 and C191 and residues C30 and C279, as typically found in chemokine receptors. Although there is yet no known ligand for GPR25-relevant clinical evidence, our assessment of GPR25 expression levels in established cancer cell lines indicated specifically high expression levels in tumor cell lines that belong to the gastrointestinal (GI) tract, out of which are the colon cancer lines HCT116 and HT29, pancreatic Panc-1 cells, and, to a lower extent, RKO cells, consistent with its property, exhibiting near-normal colon cells. In contrast, GPR25 is not found in the HTR8 ovarian cancer line, in MDA-231 and MCF-7 breast cancer cells, or in the near-normal HU epithelial line [82].

Since no known ligand for GPR25 orphan receptor was known at the time, these studies were employed. Activation of GPR25 was carried out using 10% serum. (It is acknowledged that such activation is limited and should be interpreted cautiously.) It revealed the stabilization of β-catenin and induced β-catenin transcriptional activity, as shown in HEK293 cells overexpressing *gpr25* and evaluated by Western blotting and enhanced *Lef/Tcf* activity [82]. Since GPR25 potently induces β-catenin stabilization, its possible association with LGR5 was addressed. Indeed, GPR25 co-associates with LGR5 toward markedly induced β-catenin signaling [82]. Like what was found for FZD receptors, PAR_2,_ PAR_4_, and GPR25 co-associate with LGR5. This was assessed by co-IP, immunofluorescence, and protein–protein docking analyses. It is yet unknown whether GPCR-mediated β-catenin stabilization is dependent on LGR5. This is true for the validated GPCR-LGR5, such as PAR_2_, PAR_4_, and GPR25, as well as for the predicted GPCRs, EP2, EP4, LPARs, ETAR, and PTHR1. It has been demonstrated at least for the validated GPCRs that, in the presence of LGR5, induced levels of β-catenin stabilization take place, leading to enhanced transcriptional activity. Future experimental studies may further address this issue.

**Figure 5:**
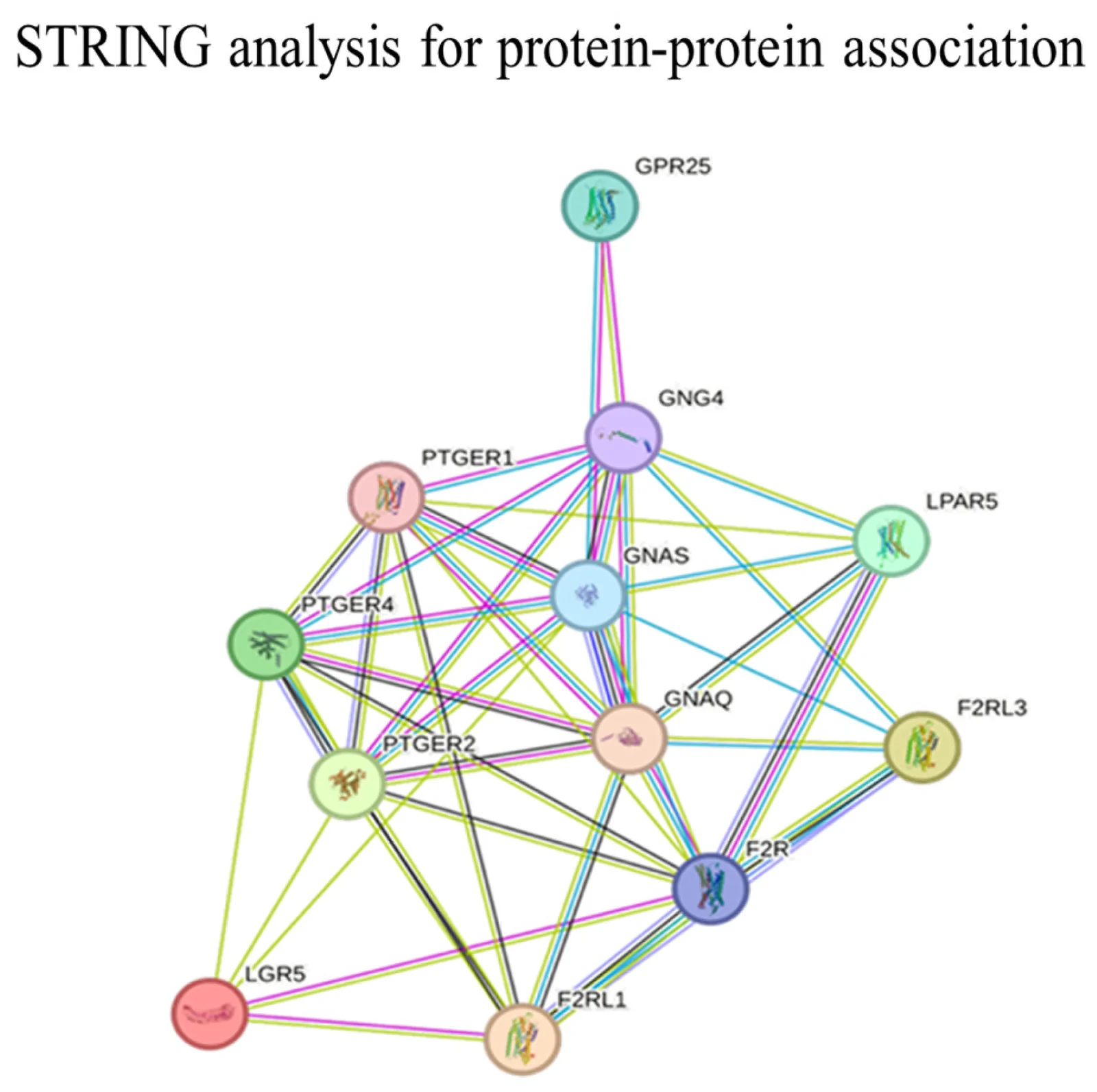
STRING-based protein–protein interaction network highlighting potential GPCR partners of LGR5. Protein–protein interaction analysis was performed using the STRING database (version 12.0) for *Homo sapiens* to explore potential associations between LGR5 and selected GPCRs and downstream signaling components. The network was generated using a confidence interaction score (0.15) to capture both known and predicted functional associations. Nodes represent individual proteins, including LGR5, GPCRs (e.g., PTGER1-4, *f2r*, *f2rl1*, *f2rl3*, LPAR5, and GPR25), and key heterotrimeric G-protein subunits (GNAS, GNAQ, and GNG4), while edges denote functional associations based on multiple sources of evidence. This figure is from the corresponding author’s laboratory (Bar-Shavit, R and associates), showing the potential association between LGR5 and selected GPCRs.

STRING analysis for protein–protein interactions between LGR5 and selected GPCRs shows the prospect of interaction between LGR5 and GPCRs, PTGER1-4, F2RL1, F2RL3, LPAR5, as well as G-protein subunits (GNAS, GNAQ, and GNG4) (Figure 5). The network illustrates potential GPCR-G protein signaling hubs that may have an impact on LGR5-related cancer stem cell pathways.

## 7. Future Perspectives

The prospect of cancer driver GPCRs as partners in stem cell functions is intriguing. Still, the molecular mechanism of GPCR involvement remains vague, awaiting an appropriate study focus. LGR5 is recognized as a widely expressed cancer stem cell protein and a significant cancer target gene. However, addressing LGR5 alone is not sufficient to eradicate cancer. Silencing of its encoding gene induces a high degree of cell plasticity such that cells that do not express LGR5 (e.g., LGR5-cells) can revert and become LGR5^+^-expressing cells and generate metastatic colonies. It is, therefore, essential to identify and allocate partners that co-associate and work in concert with LGR5 to better eliminate metastatic colonies. LGR5 and partners that can induce β-catenin stabilization may form a powerful drug-target platform hub and open new horizons for cancer therapeutic regimens.

While EP2 and EP4 receptors are expressed in colon epithelial crypt cells, no EP1 or EP3 receptor expression was observed [90,91]. Importantly, EP2 and EP4 receptors are not expressed at the normal crypt base of the colon, as opposed to their increased expression in cancer [90,91,92]. PGE2-activated EP2 and EP4 receptors have been shown to induce TOP*flash* β-catenin transcriptional activity, forming an axis of prostaglandin receptor-Gs-axin [90,92]. Regarding EP2 receptors, accumulating data shows that ALDH+, a marker of CSCs [93], is associated with breast cancer-related PGE2-linked signaling events [22,94]. Along with this line of evidence, the blocking of EP2/EP4 signaling in CSCs reduced CSC properties and decreased resistance to chemotherapy. This also led to improved chemotherapeutic efficiency in mice [39]. Therefore, it is proposed that inhibition of PGE2 signaling in breast cancer patients that block EP2/EP4 receptors reduces CSC populations and traits.

**Figure 6:**
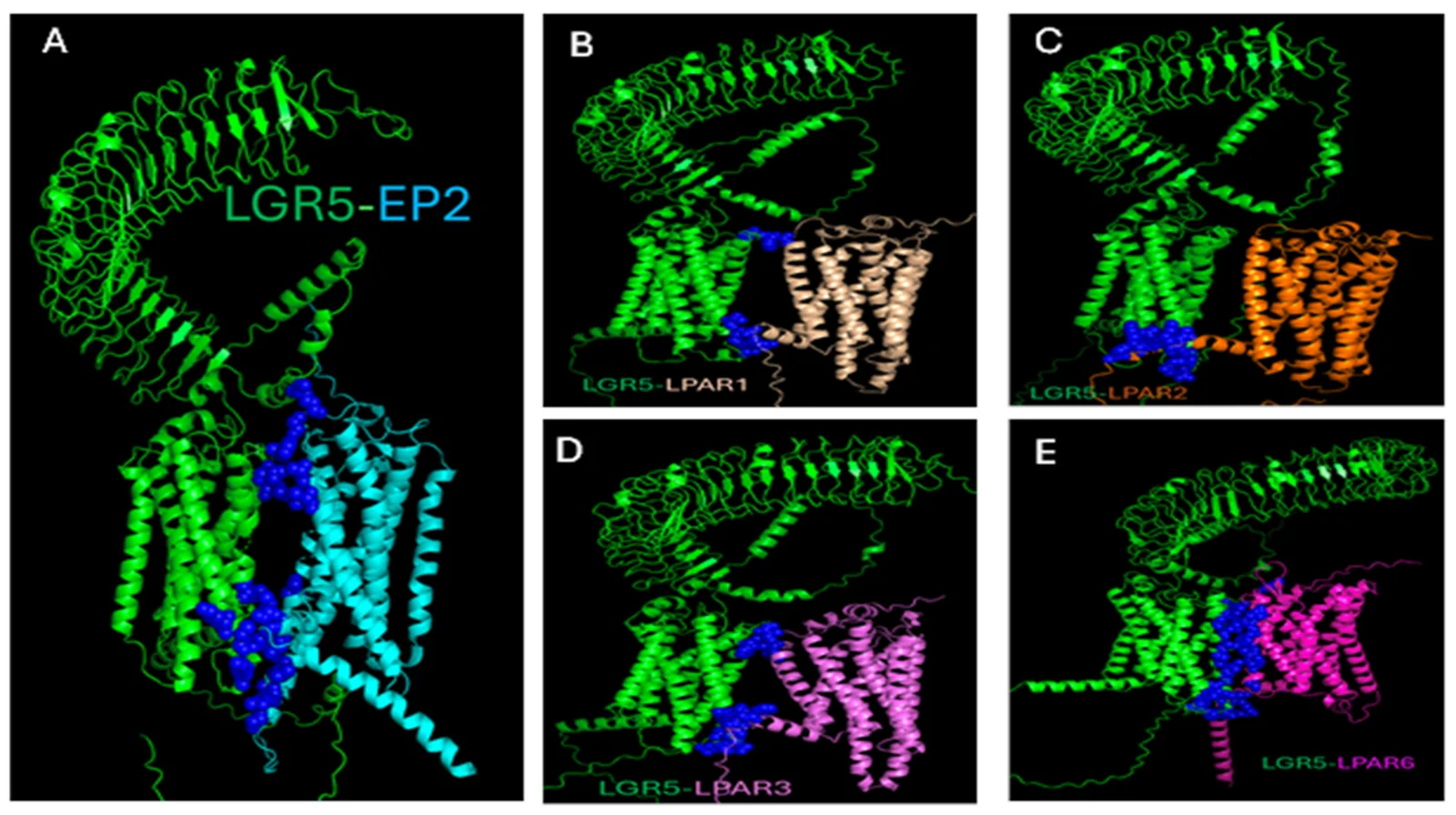
*Protein*–*protein* docking analysis using AlphaFold 3 and HADDOCKv2.4. (**A**) LGR5-EP2 strong interactions. (**B**) LGR5-LPAR1 medium docking. (**C**) LGR5-LPAR2 medium docking. (**D**) LGR5-LPAR3 weak docking. (**E**) LGR5-LPAR6 strong interactions. LGR5 is represented in green in all panels, whereas EP2, LPAR1, LPAR2, LPAR3 and LPAR6 are shown in cyan, beige, orange, light pink and magenta respectively. The predicted protein-protein interaction regions are highlighted in blue. Figure 6 is from the laboratory of Bar-Shavit R and associates, demonstrating protein-protein docking analyses between LGR5 and EP2 as also LGR5 and certain LPARs.

The lysophosphatic acid receptor 5 (LPAR_5_), a receptor for the lipid lysophosphatidic acid, is highly expressed in the intestinal epithelium as well as other members of LPAR (LPAR1-6). LPAR_5_ induces β-catenin-mediated transcriptional activity and the downstream cancer gene signature. LPARs induce β-catenin stabilization and nuclear translocation by phosphorylating GSK3β, thus inhibiting its negative regulation on β-catenin stabilization [95,96]. *lpar5* knockdown mice are associated with decreased expression of Yes-associated protein (YAP) and reduced numbers of Paneth cells. The same gene plays a role in colon regeneration after irradiation injury, maintenance of Paneth cells, as well as the niche stem cells [97]. The role of LPAR6 in tumor progression is somewhat controversial, depending on the context. LPAR6 was shown to promote hepatic cellular cancer (HCC) progression through the upregulation of *Pim3* protooncogene [98]. Some of the GPCR candidates were analyzed for the potential probability to form a complex with LGR5. Protein–protein docking analyses were employed, using the software AlphaFold 3 to predict the initial complex structures and HADDOCK 2.4 for docking and structural refinement of the candidate LGR5-GPCR complexes. PISA software version 1.52 was used to measure the following parameters: interface analysis, including interface area, interacting residues, hydrogen bonds, salt bridges, and solvation-energy gain. These analyses revealed strong interactions between LGR5-EP2 and LGR5-LPAR6. Moderate type of interactions between LGR5-LPAR2 and LGR5-LPA3 and weak interactions between LGR5-LPAR1 (Figure 6, Table 2).

Computational analysis of candidate LGR5-GPCR complexes. The computational analysis was performed in three main stages. First, initial structural models of selected LGR5–GPCR complexes were predicted using AlphaFold 3. Second, the candidate complexes were subjected to protein–protein docking and structural refinement using HADDOCK 2.4. Third, PISA was used to analyze the protein–protein interfaces, including interface area, interacting residues, hydrogen bonds, salt bridges, and solvation-energy gain. These parameters were comparatively evaluated to assess the predicted structural feasibility of the candidate LGR5–GPCR complexes.

Importantly, protein–protein docking analyses are helpful, suggesting which is a preferable co-associating candidate. It should be kept in mind that the software analyses are pending confirmation of biochemical data.

The molecular link between endothelin A receptor (ETAR) following activation by ET-1 (endothelin-1) and β-catenin stabilization was shown in ovarian [99,100] and colon cancers [101]. Therefore, ETAR is proposed to potentially serve as a regulator of CSCs niche via potential association (yet to be shown) with LGR5. PTHR1 GPCR plays a central role in bone growth, remodeling, and resorption (bone degradation). Indeed, the Wnt/β-catenin pathway is centrally involved in stem cell-based generation of the bone-forming cells osteoblasts, as well as bone cell differentiation, maturation, and osteoclast activity [102,103,104,105,106,107].

Accordingly, in a transgenic mouse model characterized by high levels of secreted Frizzled-related protein 1 (sFRP1), bone formation was inhibited, displaying the effects of attenuated PTH action on bones [103,104]. sFRP1 serves as a circulating hook capturing Wnt ligands that are no longer available for FZD receptor activation. Hence, sFRP1 overexpression enhances bone resorption. This outcome is consistent with data obtained by Diarra and colleagues [105], who found a similar result with Dkk-1, a potent Wnt antagonist, upon addressing the functional outcome of bone resorption (e.g., bone formation by osteoclasts). In addition, it was found that activation of PTHR1 leads to recruitment of LRP6 as a co-receptor [106,107], similar to the co-association of LRP6 with FZD receptors following their Wnt activation. Moreover, Romero and colleagues reported that PTH-activated PTHR1 induces β-catenin stabilization regardless of the presence of Wnts. Consequently, DVL co-associates with the PTHR1 cytoplasmic tail [107]. Overall, PTHR1 activation resembles steps in the canonical β-catenin stabilization pathway, such as the recruitment of LRP6 as co-receptor, engagement of DVL to the PTHR1 tail, and β-catenin nuclear translocation. It is therefore proposed that PTHR1 is hypothetically a strong candidate for co-association with LGR5 (pending demonstration) toward regulation of the CSCs niche.

Overall, EP2, EP4, LPAR, as well as ETAR and PTHR1, are cancer GPCRs that induce β-catenin stabilization. As such, they are potential candidates for regulating CSCs, pending further research in this direction.

## 8. Concluding Remarks

The use of LGR5 is handy and practical for detecting a wide range of solid tumors. Recently (March 2026), CDR-Life, a Swiss biotechnology company developing highly selective T-cell engagers (TCEs) to treat cancer (and some autoimmune diseases), generated the compound CDR609. CDR609 was declared as a next-generation clinical T-cell engager for the treatment of LGR5-expressing solid tumors. It shows effective killing of LGR5+ cancer cell lines co-cultured with human PBMCs (e.g., peripheral blood mononuclear cells). This is accompanied by enhanced proliferation (Ki67, a cellular marker for proliferation) and activation (CD69 and CD25, markers of T-cell activation) of cytotoxic CD8+ T cells. Overall, CDR609 kills LGR5+ cancer cells and attacks T cells. This therapy mode is projected to be capable of reaching a broad range of cancer patients presenting multiple high-incidence tumor types. Yet, challenges facing therapy based on LGR5 alone are portrayed in the plasticity phenomenon; CSCs are not static. LGR5^+^ cells drive primary tumor growth; cells can discard LGR5 to induce metastatic spreading or escape treatment, only to revert to LGR5-positive cells later. The stage of cell migration to form metastasis, which is mediated by LGR5-negative cells, is missed by targeting solely LGR5. Furthermore, upon LGR5 loss during therapy, LGR5-negative colorectal cancer cells induce the MET-STAT3 axis and resist chemotherapy-maintaining tumor growth regardless of successful LGR5 targeting.

**Scheme 1:**
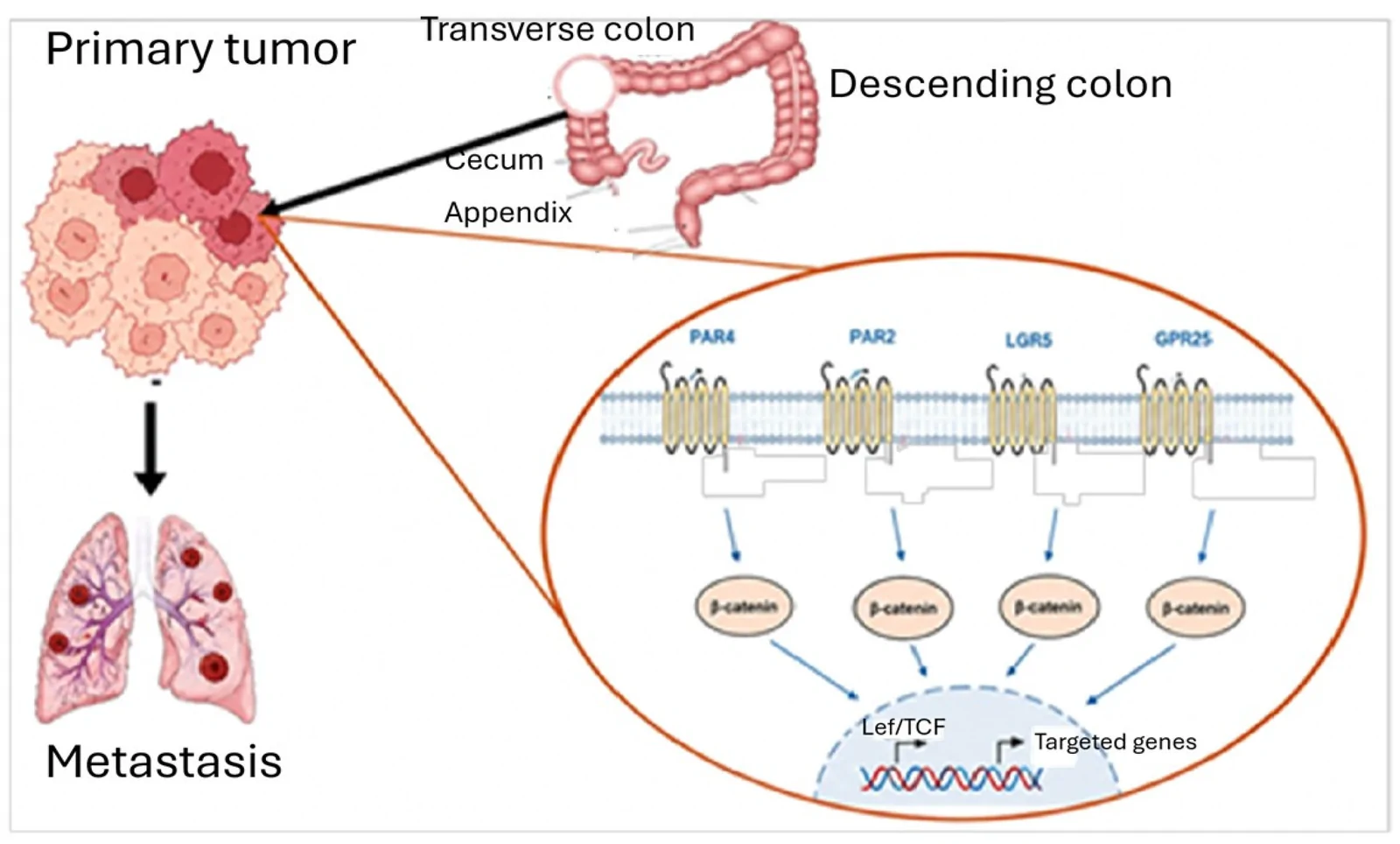
Proposed model of the GPCR-LGR5 hub in colon cancer. GPCRs that potently induce β-catenin stabilization act with LGR5 to enhance β-catenin levels and Lef/Tcf transcriptional activity. Descriptions of the colon parts are shown through transverse colon, descending colon, cecum, and appendix. The primary colon is enlarged, demonstrating metastasis in the lung. It is proposed that activation of PAR1-4 enhances β-catenin stabilization, leading to activation of transcription complex, including Lef/Tcf, for targeted gene transcription.

Lastly, one needs to bear in mind that LGR5 is a marker also of normal physiological adult epithelial stem cells. These cells oversee daily wound repair and tissue regeneration; their loss particularly affects the gastrointestinal tract. Hence, illuminating cancer GPCR as partners of LGR5 (see Scheme 1), capable of eliciting β-catenin stabilization, presents a novel opportunity to address fundamental questions regarding molecular mechanisms in the stem cell niche. This is required and necessary for finding new therapeutic regimens and novel opportunities to eradicate tumors. Overall, it is proposed that the combinatorial targeting of LGR5 and its GPCR partners may provide a more effective strategy.

## Figures and Tables

**Table 1:** GPCRs that induce β-catenin stabilization.

	Researchers	GPCR	Protease/Ligand	Functional Role	Ref.
1	Even-Ram S et al., 1998	PAR_1_	Thrombin/SFLLRN	PAR_1_ is overexpressed and activated via thrombin or SFLLRN in breast cancer.	[24]
2	Nasri I et al., 2016	PAR_2_	Trypsin/SLIGKV	PAR_2_/GSK3β axis acts to induce stem/progenitor cell survival in colon cancer.	[25]
3	Sébert M et al., 2019	PAR_1_ and PAR_2_	SFLLRN and SLIGKV	Cell survival, colony formation.	[26]
4	Strong, AA et al., 2026	PAR_2_	Trysin/SLIGKV	PAR_2_, an emerging therapeutic target.	[27]
5	Schaffner, F & Ruf, W., 2009	TF via PAR_2_	TF-VIIa	Tissue factor and PAR_2_ signaling in the tumor microenvironment.	[28]
6	Roy A et al., 2017	PAR_2_	VIIa-PAR_2_	β-catenin stabilization via AKT/GSK3β pathway in breast cancer.	[29]
7	Hiken JF et al., 2017	EP4 receptor	Prostaglandin E2 (PGE2)	EP4 is involved in cancer resistance as well as a driver of CSCs. This is reversed by GW627368x.	[32]
8	Kundu N et al., 2014	EP4 receptor	PGE2	EP4 is highly expressed in stem-like cells. CSCs are reduced following treatment with EP4 antagonists.	[33]
9	Ching McM et al., 2020	EP2 and EP4 receptors	PGE2	Cox2 enzyme is overexpressed in epithelial cancers. It catalyzes the metabolism of arachidonic acid, resulting in PGE2. PGE2 activates EP2 and EP4 involved in CSCs.	[34]
10	De Paz Linares et al., 2021	EP4	COX2/EP4	EP4 plays a role in breast cancer stemness. EP4 antagonist. Antagonists of EP4 in combination with checkpoint inhibitors are effective breast cancer therapy.	[35]
11	Walker OL et al., 2022	EP2 and EP4	PGE2	Breast cancer stem cell markers CD44+/CD24-/ALDH are high when PGE2 is high. Anti-PGE2 therapies are proposed for breast cancer.	[36]
12	Lynch JR & Wang JY, 2016	EP2 and EP4	PGE2	In breast cancer, the EP4 antagonist RQ-15986 is useful in therapy.	[37]
13	Wang D et al., 2015	EP2 and EP4	PGE2	PGE2 promotes CSC growth in colon cancer via EP4-PI3K and NF-κB.	[38]
14	Lin M-C et al., 2018	EP2 and EP4	PGE2	COX2/PGE2/EP4 signaling induces basal-like breast cancer chemoresistance. EP4 antagonist enhances extracellular vesicle formation, which promotes a non-CSC state. Increased chemosensitivity acts to reduce tumor burden.	[39]
15	Minematsu H et al., 2022	EP2 and EP4	PGE2	PGE2 is high and active in CSCs. It acts via the AKT/PI3K axis.	[40]
16	Hirata N et al., 2022	LPAR3	LPA	Triple-negative breast cancer (TNBC) stem cells express high ALDH through LPAR3. LPAR3 antagonist inhibits ALDH-expressing cells. It acts via transient receptor potential cation channel C3 (TRPC3), promoting the LPA–TRPC3 axis.	[41]
17	Seo EJ et al., 2016	LPAR1	LPA	In spheres from ovarian cancer (OC) patients and cell lines, LPA is high, as are levels of OCT4, SOX2, ALDH, and autotaxin. Autotaxin knockdown inhibited LPA-tumors and drug resistance. Silencing AKT1 suppressed CSCs. Autotaxin–LPA–LPAR–AKT1 axis.	[42]
18	Nadhan H et al., 2025; Xiang, H et al, 2020	LPAR1-6	LPA	LPA is synthesized in cancer cells. LPA signaling promotes chemoresistance. In OC, LPA promotes the expression of ZIP4 and elicits upregulation of EGFR and, thus, resistance to cisplatin and Dox.	[43,44]
19	Karalis, T et al.	LPAR1-6	LPA	LPA as an oncometabolite.	[45]
21	Wu, P.Y et al.	LPAR1-6	LPA	LP-mediated lymphangiogenesis in prostate cancer.	[46]
22	Balijepalli, P et al.	LPAR1-6	LPA	LPA signaling in cancer cells.	[47]
23	Xu, Y	LPAR1-6	LPA	LPA signaling in ovarian cancer.	[48]
24	Pyne, N. J et al.	SIPR1-4	SIP	SIP signaling in cancer.	[30]
25	Patmanathan, SN et al.	SIPR1-4	SIP	Mechanism of SIPR signaling in cancer.	[31]

**Table 2:** Comparative analysis of predicted LGR5-GPCR protein-protein interactions using AlphaFold 3 and HADDOCK v 2.4. Table 2 is from the laboratory of Bar-Shavit R and associates, demonstrating parameter evaluations between LGR5 and EP2 as also LGR5 and certain LPARs.

Rank	Candidate Complex	No. of Interface Residues (LGR5/GPCR)	Interface Area (A^2^, LGR5/GPCR)	Solvation Energy Gain (kcal/mol, LGR5/GPCR)	H-Bonds	Salt Bridges	Predicted Structural Feasibility
1 (tie)	EP-LGR5	25/27	862.5/833.3	−11.8/−11.8	3	0	High
1 (tie)	LPAR6-LGR5	25/27	862.5/833.3	−11.8/−11.8	3	0	High
3	LPAR3-LGR5	24/22	694.1/728.3	−7.8/−8.3	4	1	Moderate
4	LPAR2-LGR5	14/17	453.3/456.7	−4.2/−4.4	2	5	Moderate
5	LPAR1-LGR5	14/11	372.9/412.9	−3.3/−1.1	5	2	Low

**Note:** Predicted structural feasibility was assigned by comparative evaluation of interface size, number of interface residues, solvation-energy gain, hydrogen-bond interactions, and salt bridges among the candidate complexes. Complexes exhibiting larger interaction interfaces and more favorable solvation-energy gains together with supporting intermolecular contacts were classified as High, intermediate profiles as Moderate, and comparatively weaker interfaces as Low. These classifications represent computational predictions and require experimental validation.

## Data Availability

No new data were created or analysed in this study. Figure 5 is from the corresponding author’s laboratory (Bar-Shavit, R and associates), showing the potential association between LGR5 and selected GPCRs. Figure 6 and Table 2 are from the laboratory of Bar-Shavit R and associates, demonstrating *protein-protein* docking analyses and parameter evaluations between LGR5 and EP2 as also LGR5 and certain LPARs. Data sharing is not applicable to this article.

## Abbreviations

CSCs	Cancer stem cells
GPCRs	G protein-coupled receptors
PAR	Protease-activated receptor
LGR5	Leucine-rich repeat-containing G protein-coupled receptor 5 (also LGR4 and LGR 6)
GPR25	G protein-coupled receptor 25
EP2	Prostaglandin receptor 2 (also EP4)
LPA	Lysophosphatidic acid receptor
CBC	Crypt-based columnar
FZD	Frizzled receptor
RNF43	Ring finger 43
ZNRF3	Zinc and ring finger 3
ECM	Extracellular matrix
TME	Tumor microenvironment
TCGA	The Cancer Genome Atlas
SIP	Sphingosine phosphate
LRP6	LDL receptor-related protein 6
DVL	Dishevelled
GSK3β	Glycogen synthetase kinase 3β
RSPO	RSpondin
IQGAP1	IQ motif containing GTPase-activating protein 1
PH-domain	Pleckstrin homology domain
EGFR	Epidermal growth factor receptor
p-Tyr	Phospho tyrosine
CXCL17	Chemokine ligand 17
GI	Gastrointestinal
PTHR1	Parathyroid receptor 1
ETAR	Endothelin receptor A
ETBR	Endothelin receptor B
YAP	Yes-associated protein
